# Short-term effectiveness of a community health worker intervention for HIV-infected pregnant women in Tanzania to improve treatment adherence and retention in care: A cluster-randomized trial

**DOI:** 10.1371/journal.pone.0181919

**Published:** 2017-08-31

**Authors:** Nerissa Nance, Prosper Pendo, Joseph Masanja, David Paul Ngilangwa, Karen Webb, Rita Noronha, Sandra I. McCoy

**Affiliations:** 1 School of Public Health, University of California, Berkeley, California, United States of America; 2 Ministry of Health, Community Development, Gender, Elderly, and Children (MOHCDGEC), Dar es Salaam, Tanzania; 3 Amref Health Africa, Dar es Salaam, Tanzania; 4 Organisation for Public Health Interventions and Development (OPHID), Harare, Zimbabwe; 5 Jhpiego, Baltimore, Maryland, United States of America; Azienda Ospedaliera Universitaria di Perugia, ITALY

## Abstract

**Introduction:**

Community health workers (CHWs) are lay workers who have the potential to enhance services to prevent mother-to-child HIV transmission (PMTCT) and improve the health of women living with HIV infection. We conducted a cluster-randomized trial of an intervention to integrate CHWs with ‘Option B+’ PMTCT services in Shinyanga Region, Tanzania.

**Methods:**

The intervention was implemented for 11 months and included four integrated components: 1) formal linkage of CHWs to health facilities; 2) CHW-led antiretroviral therapy (ART) adherence counseling; 3) loss to follow-up tracing by CHWs; and 4) distribution of Action Birth Cards (ABCs), a birth planning tool. We cluster-randomized 32 facilities offering PMTCT services, within strata of size, to the intervention (n = 15) or comparison (standard of care, n = 17) groups. Intervention effectiveness was determined with a difference-in-differences strategy based on clinical and pharmacy data from HIV-infected postpartum women at baseline (births in 2014) and endline (births April-Oct 2015). The primary outcome was retention in care between 60 and 120 days postpartum. Secondary outcomes included ART initiation, timing of ART initiation (as measured by week of gestation), and ART adherence 90 days postpartum, measured using the medication possession ratio (MPR≥95%).

**Results:**

Intervention and comparison facilities were similar at baseline. Data were collected from 1,152 and 678 mother-infant pairs at baseline and endline, respectively. There were no significant differences in retention in care, ART initiation, or timing of ART initiation between the intervention and control groups. Adherence (MPR≥95%) at 90 days postpartum was 11.3 percentage points higher in the intervention group in ITT analyses (95% CI: -0.7, 23.3, p = 0.06), though this effect was attenuated after adjusting for baseline imbalance (9.5 percentage points, 95% CI: -2.9, 22.0, p = 0.13). Among only sites that had the greatest fidelity to the intervention, however, we found a stronger effect on adherence (13.6 percentage points, 95% CI: 2.5, 24.6).

**Conclusions:**

Despite being feasible and acceptable, the CHW-based intervention did not have strong effects on most PMTCT indicators. CHW involvement in PMTCT programs may improve ART adherence among HIV-infected postpartum women, however, and success appears heavily dependent on program implementation.

**Trial registration:**

Registry for International Development Impact Evaluations (RIDIE, ID 552553838b402) and ClinicalTrials.gov (NCT03058484)

## Introduction

In 2008, the United Nations Programme on HIV/AIDS (UNAIDS) identified 22 countries that accounted for 90% of pregnant women in need of prevention of mother-to-child HIV transmission (PMTCT) services and targeted them for intensive support.[1] This included promoting the scale up of Option B+, a strategy first recommended by WHO in 2013 that places pregnant, HIV-positive women on lifelong antiretroviral therapy (ART), regardless of clinical or immunological stage.[2,3] From 2009 to 2014, vertical transmission rates in the 22 priority countries declined by 48%,[1] a significant milestone that nevertheless leaves room for further reduction. Tanzania exemplifies this situation, as mother-to-child transmission (MTCT) accounted for one of every 5 new HIV infections in 2014, despite major declines in MTCT in the past decade.[4]

Antenatal care, HIV testing, linkage to HIV care and treatment, and adherence to ART are the essential series of services to prevent mother-to-child transmission of HIV. This ‘PMTCT cascade’ can reduce transmission to between 2% and 5%.[5] The highest risk of transmission is observed among women who missed clinic visits or dropped out of PMTCT care.[6] The impact of poor retention in care is most acute in settings with a high HIV burden, where a small proportion of women disengaging from care can translate to a large number of infected infants.[7] Despite significant improvements in the uptake of services along the cascade, retention and adherence among pregnant and postpartum women remains a significant challenge across sub-Saharan Africa. Loss to follow up (LTFU) rates differ dramatically by context, varying between 19% to close to 90% from one to six months postpartum, with the greatest loss from the cascade occurring in the first year postpartum.[8]

The effectiveness of interventions to improve retention in care among HIV-infected pregnant women is mixed. A recent systematic review suggests evidence is generally weak, though in part due to study quality.[9] Furthermore, challenges retaining women in the PMTCT cascade must also be viewed in light of the broader healthcare workforce shortage that affects much of sub-Saharan Africa.[10–12] The success of Option B+ may be threatened by overburdened clinics and staff that in turn affect service distribution, quality, and the patient experience.[10] Consequently, paraprofessionals, like community health workers (CHWs), are emerging as key partners in the delivery and/or enhancement of health services in the community. A new and growing body of evidence supports the effectiveness of CHW task-shifting/sharing in PMTCT programs, particularly given their ability to aid with linkage to and retention in care and adherence.[13–15] The present study examines the effectiveness of one such intervention in the Tanzanian context. Tanzania is one of UNAIDS’ 22 priority countries for PMTCT support and faces health workforce scarcity; Tanzania has one physician per 125,000 people, and is among the 10 countries with the lowest density of nurses and midwives.[16] Approximately 12,000 CHWs provide key maternal, newborn, and child health (MNCH) services to women and families across the country.[17] These part-time volunteer workers are elected by village councils and their community influence make them unique allies in the provision of key health services. This trial is the first of its kind to examine whether a community-level CHW intervention can improve short-term retention in care and adherence to ART among pregnant women living with HIV infection in the context of Option B+ in Tanzania.

## Methods

### Study design

We conducted a cluster-randomized controlled trial to evaluate the short-term effectiveness of a CHW intervention to improve retention in care and ART adherence for HIV-infected pregnant and postpartum women.[18] The intervention was implemented between May 2015 and March 2016 by Amref Health Africa in selected communities in Shinyanga Region, Tanzania. Shinyanga is a resource-constrained landlocked region in the northwest of Tanzania, where HIV prevalence is 7.4%.[19] The project was intended to evaluate the intervention’s short-term effectiveness during an 18-month study period. Baseline data were collected between July and December 2015; the baseline cohort of women included HIV-infected women who were 90 days postpartum between April 1, 2014 and March 31st, 2015. Endline data were collected between January and May 2016; the endline cohort included HIV-infected women who were 90 days postpartum between July 1, 2015 and January 31, 2016. The intervention period was 11 months in length. This study was approved by the Amref Health Africa Institutional Review Board in Tanzania as well as the Committee for the Protection of Human Subjects at the University of California, Berkeley. The protocol for this study was pre-registered with the Registry for International Development Impact Evaluations (RIDIE, ID-552553838b402).

### Community eligibility criteria

Communities were selected for the study if they were served by health facilities meeting the following criteria: 1) the provision of on-site HIV treatment and care services, including ART; 2) the provision of PMTCT services and reproductive and child health services; 3) the facility was not participating in another study or program linking CHWs to PMTCT; and 4) the facility managed a caseload of ≥75 female patients on ART in 2014. Thirty-seven of 56 communities (with their associated health facilities) in the Shinyanga region met these inclusion criteria for the study.

### Randomization procedures

**Fig 1:**
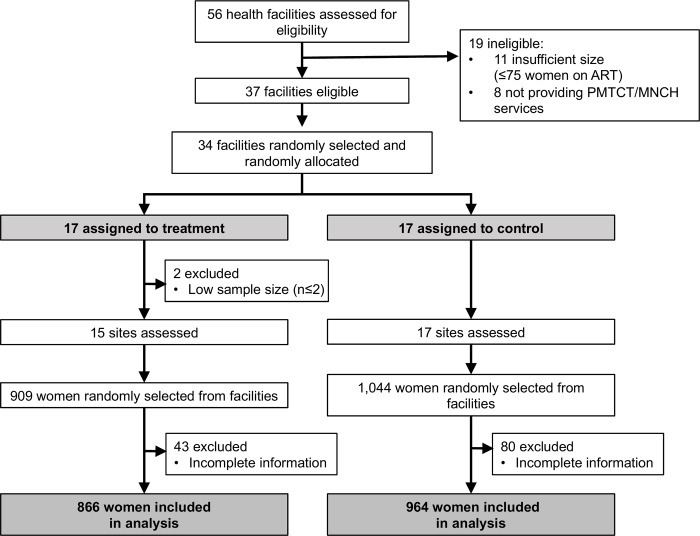
Trial profile.

We randomly selected 34 of the 37 eligible communities for the study within two strata defined by patient caseload (greater than or equal to 550 female ART patients in 2014). Randomization procedures were conducted at the University of California, Berkeley using a random sequence generator in STATA.[20] After stratifying by the size of the community’s health facility, the research team assigned communities in a 1:1 ratio to treatment and control groups. Two sites were excluded post-randomization due to lower than expected caseloads (<2 HIV-positive pregnant or postpartum women in care in 2014), leaving 32 sites (15 intervention and 17 control) for the primary analysis (Fig 1).

### Intervention description

The intervention was implemented in the communities located in intervention facility catchment areas (the surrounding geographical area served by the facility), and was comprised of four components: 1) formal linkage of CHWs to health facility staff for mentorship and supportive supervision, 2) the distribution of “Action Birth Cards” by CHWs and health facilities to pregnant and postpartum women, 3) the provision of ART adherence counselling to pregnant and postpartum women living with HIV infection by CHWs, and 4) CHW tracing of women who defaulted from HIV care. Formal linkage of CHWs to the health facility was intended to further integrate CHWs into the formal health system, and involved weekly supportive supervision meetings with facility staff to discuss caseloads and challenges. The Action Birth Card is an interactive birth planning tool that reminds women about antenatal care visits, helps them plan for delivery, and if HIV-positive, encourages them to receive necessary care. The Action Birth Card was originally developed in Zimbabwe where it has demonstrated preliminary effects on service utilization.[21] The study adapted the Action Birth Card to Tanzania’s context and translated it to Kiswahili. The card was distributed to pregnant women at health facilities and in their homes during the intervention period. ART adherence counselling training was provided to all CHWs in the treatment catchment areas in April 2015, where they learned methods to support patients to overcome barriers to ART adherence. The training also included orientation to the Ministry of Health, Community Development, Gender, Elderly, and Children (MoHCDGEC) defaulter tracking tool for health staff and CHWs. Using this tool, CHWs in intervention facilities worked with healthcare workers to identify and locate pregnant and postpartum HIV-positive women who had fallen out of antenatal and/or HIV care with the goal to re-link them back to care. Together, these four strategies were intended to integrate community-based health services delivered by CHWs with HIV prevention, treatment, and care—bridging the gap between community and facility, and enhancing the potential benefits of Option B+.

### Outcomes

The outcomes were measured among HIV-infected pregnant and postpartum women attending facilities in study communities and were linked to the series of services on the PMTCT cascade. The primary outcome was retention in care 90 days postpartum. Retention in care was calculated both among the full sample and among women with prior evidence of HIV clinic care (i.e., women assigned an HIV clinic identification number). Women were considered retained in care at 90 days postpartum if they had an HIV clinic care visit or pharmacy refill between 60 to 120 days postpartum (inclusive).

Secondary outcomes were also measured among women attending health facilities and included: initiation of ART during pregnancy, timing of ART initiation, and ART adherence 90 days postpartum. Initiation of ART was measured as the number of HIV-infected women in the sample who had any evidence of beginning ART after pregnancy, among women without evidence of treatment prior to pregnancy (women who had initiated ART before the current pregnancy were excluded). The timing of ART initiation during pregnancy was defined by gestational week at start of ART, which was computed using standard approaches (i.e., 40 weeks prior to the date of birth or, when available, the expected delivery date based on last menstrual period). ART adherence was measured using the medication possession ratio (MPR), a pharmacy-based measure of adherence defined as the proportion of days in an interval when ART was prescribed and individuals are in possession of ≥1 dose of ART (determined with pharmacy dispensing data).(22) MPR is associated with short-term virologic outcomes.[22–25] We computed MPR overall (continuous scale) and determined the proportion of women with MPR ≥95% and MPR≥80% from birth to 90 days postpartum, consistent with prior literature about the adherence threshold associated with virologic suppression.[26]

### Data collection

**Fig 2:**
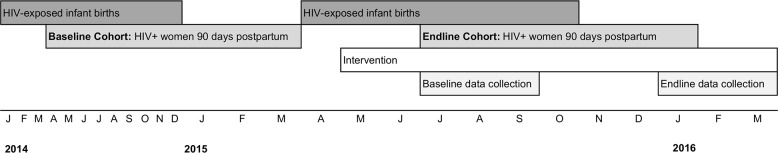
Baseline and endline cohort timeline and data collection periods for the impact evaluation of a CHW-based intervention for PMTCT in Tanzania, 2014–2015.

To examine the effectiveness of the intervention, we used a ‘difference-in-differences’ approach, whereby the effect of the intervention on the outcome can be ‘differenced out’ from time-constant effects.[27] Thus, we collected data from two cohorts of women attending both intervention and control facilities. The baseline cohort included HIV-infected women who delivered an infant between January and December 2014 (90 days postpartum April 2014 to March 2015); these women were unexposed to the intervention. The endline cohort included HIV-infected women who delivered an infant between April and October 2015 (90 days postpartum July 2015 to January 2016; Fig 2); these women were both exposed and unexposed to the intervention (depending on their community’s study arm). We extended the timeline of the baseline cohort to one year to increase sample size, but were unable to do so in the endline cohort because of the short length of the intervention. Women were considered eligible for inclusion in the study if they were identified in one of the medical registers used for sampling at the facility (described below), were HIV-positive, and had a child born in either the baseline or endline cohorts. Women in the endline cohort who attended a facility in the intervention group were exposed to the intervention for 4 to 9 months of their pregnancy and 90 days postpartum, depending on their delivery date. We hypothesized that this exposure would be the minimum effective intervention ‘dose’, given that, on average, women in Tanzania are 5.4 months pregnant at their first ANC visit when they have the initial opportunity to begin ART, if they haven’t already.[19]

At baseline and endline, the impact evaluation team visited the 32 facilities and abstracted data from facility records. At each site, individuals were identified for inclusion through the complete enumeration of HIV-positive women meeting the inclusion criteria in facility registers. We selected all eligible women in the two cohorts, with the exception of large facilities (defined as ≥50 eligible women at baseline or endline), where we selected a systematic random sample of eligible women using a pre-determined sampling fraction based on facility size (e.g., 0.75 for facilities with 51–100 patients, inclusive, and 0.50 for facilities with 101–200 patients). We abstracted patient-level data from various medical registers that are a central repository for patient medical information (Antenatal Care Register, Postnatal Register, etc.) as well as data from individual patient records used for HIV patient care.

In addition to the facility records, the team also collected baseline and endline facility surveys that detailed the size, area and scope of services available at each facility. All data from the facility records and the facility survey were recorded electronically by the study team on tablet computers using Qualtrics survey software.[28]

### Statistical analysis

We first described characteristics of the facilities and study participants and examined the data for baseline imbalances after randomization. We then examined differences in the outcomes in the control group over time, to delineate temporal trends in the absence of the intervention.

For the primary and secondary outcomes, we followed the ‘difference-in-differences’ analysis approach,[27] where we first constructed unadjusted intent-to-treat linear regression models using indicator variables for both time (baseline, endline) and treatment status (intervention, control), and an interaction term equal to the product of time and treatment status. In these models, the coefficient of the interaction term between time and treatment status is equal to the effect estimate between treatment and control groups, controlling for changes observed over time.[27] The outcome was on a binary scale for the primary outcome of retention in care and MPR≥95%, MPR≥80%, and ART initiation (up to 90 days postpartum); to verify the appropriateness of using a linear probability model on a binary outcome, we examined the marginal probabilities of the outcomes to confirm they did not take values outside of zero and one. Though we checked the assumptions necessary to use a linear probability model for a binary outcome[29], we additionally re-ran the analyses with a logistic regression model for the outcomes of retention in care, adherence (MPR≥95% and MPR≥80%), and ART initiation. We found that the conclusions did not significantly change (S1 Table). The outcome was on a continuous scale in the case of timing of ART initiation.

Secondary analyses included repeating the above analyses, adjusting for factors imbalanced at baseline, and conducting a sensitivity analysis to explore differences in treatment effect by how well the intervention was implemented. To achieve this, we created a summary score for each community that represented the average “fidelity” score associated with each of the four intervention components, using monitoring data. Thus, to measure the linkage of CHWs and health workers, we used the average number of recorded meetings between CHWs and health care workers per month at each facility. For the Action Birth Card, we used the number of cards distributed by the facility, divided by the number of HIV+ women that were recorded as visited by CHWs (to account for differing population sizes). To measure adherence counselling, we used the average number of CHW visits per woman at each site (from CHW log forms). For tracing defaulters, we used the proportion of defaulters successfully traced at the facility relative to the total number of defaulters at the site. We ranked each of these intervention component scores (from 1 to 15, 1 being the lowest rank and 15 being the highest rank) and then averaged them to create a summary score. The resulting average score across the four components was then analysed as part of a three-level treatment variable, grouped into “high performing” (above the median) sites, “low performing” (at or below the median) sites, and control sites.

All analyses were weighted by facility to account for: 1) the probability of facility selection within strata of large and small sites, 2) the probability of individual selection given the number of eligible women at each site, and 3) the response rate (number of records with adequate identifying information to connect observations across registers; 10.5% of initial baseline sample excluded). Standard errors were adjusted for the clustered nature of the data through the svy command in STATA.[30]

### Sample size

The study size was determined for the primary outcome of retention in care 90 days postpartum. In our preliminary analysis of pregnant women in Shinyanga, we found that 36% of HIV-infected pregnant women who initiated ART were lost within 100 days after delivery.[31] Thus, we assumed that in the control communities, 67% of women would be retained in care at 90 days postpartum. With an ICC of 0.04 and the assumption that the intervention would increase the proportion of women who are retained in care postpartum from 67% to at least 80% (detectable effect size of 13 percentage points, or 20%), we required at least 39 observations per community to have 80% power to reject the null hypothesis of no effect. We calculated the observed ICC of the final sample using STATA’s loneway command.[32]

## Results

Of the 32 facilities included in the study, 17 were assigned to the control group and 15 to the treatment group (Fig 1). Data were retrospectively collected from facility registers on 1,152 women at baseline and 678 women at endline; at endline this corresponded to 909 women in the treatment group and 1,044 women in the control group. A total of 123 women (10.5%) were excluded from the baseline sample because of incomplete information; this exclusion was not significantly different across treatment group (Table 1). Overall, treatment and control groups were similar at baseline, with the exceptions of average number of CHWs per facility and the prevalence of early infant testing (15.2 vs. 9.2 CHWs per facility; 37.6% vs. 28.9% of HIV-exposed infants tested, respectively, Table 1).

**Table 1:** Baseline site-level characteristics, by study arm, Tanzania, 2014^a^^,^^b^.

	Treatment sites Mean (SE^c^)	Control Sites Mean (SE)	p-value
Total number of sites	15	17	—
**Facility characteristics**			—
Type of facility (%)			0.27
Government health center	53.2	76.2	
Government dispensary	25.8	23.8	—
Religious/voluntary hospital	6.4	0	—
Religious/voluntary health center	14.6	0	—
Estimated population in catchment area	16321(1895)	17015 (2896)	0.84
Number of staff	16.0 (1.7)	18.8 (3.4)	0.50
Average number of CHWs per site mean (SE)	15.2 (2.6)	9.2 (1.0)	0.03**
Average number of CHWs per capita^d^	0.07	0.13	0.06*
Frequency of CHW meetings (%)			0.60
1–2 times/month	0	6.4	—
3–4 times/month	34.5	35.6	—
>4 times/month	65.6	58.0	—
**Service provision**			—
Number of days per week ANC services are available (mean (SE))	4.2 (0.4)	4.9 (0.3)	0.14
Provision of ART services, including Option B+ services (%)	100	100	—
Provision of postnatal services (%)			0.35
At facility	100.0	94.4	—
Referral to another facility	0	5.6	—
Provision of labor and delivery services (%)			0.25
At facility	83.3	100.0	—
Referral to another facility	11.3	0	—
Both	5.6	0	—
Adherence counseling provided (%) Every ANC visit	38.7	33.7	0.95
Every CTC visit	54.9	60.6	—
Treatment initiation only	6.4	5.6	—
**Other attributes**			—
Mean number of missing women from baseline sample^e^	4.7 (1.5)	2.7 (0.62)	0.12
Early infant HIV diagnosis by 6–18 months postpartum (% among HIV-exposed infants)	37.6 (3.4)	(1.9)	0.03**

** Significant at the α = 0.05 level

* Significant at the α = 0.10 level

a) Estimates are weighted by site size (large vs. small)

b) Statistical significance of differences between control and treatment groups determined using chi-square tests for categorical variables and t-tests for continuous variables

c) SE = standard error

d) To calculate average number of CHWs per capita, the number of CHWs at the site was divided by the estimated population in the catchment area

e) Women were excluded from the sample if they did not have necessary identifying information to link them across records (n = 123)

### Control group changes over time

In the control group, we observed significant improvements over time in retention in care (46.4% vs. 58.8%; difference: 12.6; 95% confidence interval CI: 4.5, 20.8), the timing of initiation of ART (27.3 vs. 24.3 weeks; mean difference: -3.0 weeks; 95% CI: -5.2, -0.8), and MPR≥80 (34.9 vs. 41.6 percent; difference 6.7, 95% CI: 1.5, 11.9). No changes in MPR≥95 were observed in the control group over time (difference: 1.6, 95% CI: -2.9, 6.1 for MPR≥95; Table 2).

**Table 2:** Baseline and endline outcomes among HIV-positive pregnant and postpartum women in Tanzania, stratified by treatment group, 2014–2015^a^.

		Control Group			Treatment Group				
	N	Baseline	Endline	Difference^j^	Baseline	Endline	Unadjusted DiD^h^ (CI^i^)	Adjusted DiD^h^ (CI^i^)		
**Total**	1830	590	374	—	562	304	—	—		
**Women retained in care (%)**^**b**^^,^^**c**^	1830	34.6 (2.6)	47.3 (5.6)	12.7 (0.6, 24.7)**	35.3 (1.9)	45.3 (4.6)	-2.7 (-16.3, 10.9)	-4.5 (-17.5, 8.6)		
Full Sample										
**Women retained in care (%)**^**b**^^,^^**c**^	1348	46.4 (2.6)	59.0 (4.4)	12.6 (4.5, 20.8)**	47.9 (3.0)	65.6 (3.4)	5.0 (-5.9, 15.9)	0.9 (-11.8, 13.7)		
Women with evidence of care										
**Women initiating ART**^**d**^	1544	55.1 (4.8)	46.0 (3.5)	-9.1 (-16.9, -1.3)**	46.1 (3.3)	45.1 (3.6)	8.0 (-2.3, 18.4)	5.9 (-2.4, 14.2)		
**Timing of ART initiation**^**e**^ **(mean, SE**^**i**^**)**	787	27.3 (0.6)	24.3 (1.1)	-3.0 (-5.2, -0.8)**	27.2 (0.8)	26.7 (1.3)	2.5 (-1.6, 6.5)	2.0 (-2.0, 6.0)		
**Women with MPR≥95%(%)**^**f**^^,^^**g**^	820	21.2 (3.1)	22.8 (3.1)	1.6 (-2.9, 6.1)	16.2 (3.8)	29.1 (4.8)	11.3 (-0.7, 23.3)*	9.5 (-2.9, 22.0)		
**Women with MPR≥80% (%)**^**f**^^,^^**g**^	820	34.9 (4.4)	41.6 (4.2)	6.7 (1.5, 11.9)**	27.8 (4.1)	46.7 (5.9)	12.2 (-0.7, 25.1)*	8.9 (-3.2, 21.1)		

* significant at the α = 0.10 level

** significant at the α = 0.10 level

a) Estimates were weighted for selection, site size and, at baseline, missing women

b) Women retained 0–90 days postpartum, calculated for n = 1830 all women in the sample, and n = 1348 women with evidence of HIV care

c) Observed intraclass correlation coefficient (ICC) for primary outcome of retention in care = 0.04

d) Number of women initiating ART calculated for the n = 1544 women who did not have evidence of beginning ART prior to pregnancy; women were considered to have initiated ART if they had any evidence of ART use between pregnancy and 90 days postpartum

e) ART initiation timing calculated for n = 787 women who had an ARV start date on record, and who began ART after pregnancy; women were considered to have initiated ART if they had any evidence of ART use between pregnancy and 90 days postpartum

f) MPR ≥95 (and MPR≥80) are the women that have 95% adherence (or 80% adherence) or greater according to the medicine possession ratio (MPR) calculation, defined as the number of pill days dispensed over the number of days elapsed from the infant’s birth to 90 days postpartum

g) MPR calculated for n = 820 women who had complete ARV dispensing information

h) DiD = difference-in-differences estimate, generated through the linear regression of the outcome with an interaction variable for time and treatment status (see methods); adjusted model additionally adjusted for factors imbalanced at baseline (HEID testing and number of CHWs at the facility)

i) SE = standard error; CI = confidence interval

j) Difference between measurements at baseline and endline in control group

### Intervention effectiveness on retention in care

The intervention had little to no effect on retention in care at 90 days postpartum. In the intent-to-treat (ITT) analysis of the full sample (n = 1830), we found a non-significant -2.7 percentage point decrease in 90-day retention between treatment and control groups, controlling for time-constant effects (95% CI: -16.3, 10.9). Among women with evidence of HIV care (n = 1348), we found a non-significant 5.0 percentage point increase in retention at 90 days postpartum comparing treatment to control (95% CI: -5.9, 15.9; Table 2). These results were attenuated after adjusting for factors that were imbalanced at baseline (Table 2).

### Intervention effectiveness on secondary outcomes

The intervention had little to no effect on secondary outcomes in the ITT and adjusted analyses. Among women who did not begin ART prior to pregnancy, there was an 8.0 percentage point increase in ART initiation in the treatment group compared to the control group over time (95% CI: -2.3, 18.4). Those in the treatment group also started ART on average 2.5 weeks earlier in their pregnancy compared to those in the control group (95% CI: -1.6, 6.5), although neither of these results was statistically significant. The proportion of HIV-infected women achieving at least 95% MPR was 11.3 percentage points greater than women in control sites (95% CI: -0.7, 23.3). The proportion of HIV-infected women achieving at least 80% MPR also increased by 12.2 percentage points in the intervention group compared to the control group (95% CI -0.7, 25.1; Table 2). After adjustment for baseline imbalances, effect estimates were attenuated across all indicators (Table 2).

### Heterogeneity of treatment effect by intervention fidelity

The community-level intervention fidelity score ranged from 5.1 to 13.1 before dichotomization. Seven sites were classified as ‘high’ fidelity (above the median rank score of 8), and the remaining 8 were classified as low-fidelity (at or below the median). We found evidence of heterogeneity of intervention impact for the percent of women initiating ART during pregnancy, MPR≥95, and MPR≥80. In unadjusted analyses, the sites with a “high” treatment fidelity score had more women initiating ART during pregnancy compared to the control sites (14.4 percentage point difference; 95%CI: 4.9, 23.8, Table 3), whereas those with a low score had little difference with control sites (2.7%; 95% CI: -10.0, 15.3). Also in facilities with high fidelity to the intervention, MPR≥95 was 14.3 percentage points higher than control sites (95% CI: 2.0, 26.6), whereas there was no effect of the intervention in low fidelity sites compared to the control group (8.6 percentage points, 95% CI: -9.2, 25.5). This pattern was similar for MPR≥80; high-fidelity facilities had 12.6 percentage points more women achieving MPR≥80 compared to the control group (95% CI: -2.4, 27.5), whereas low-fidelity facilities had a non-significant 11.0 percentage points more women with MPR≥80 compared to the control group (95% CI: -6.8, 28.7; Table 3). Scores for MPR≥80 and MPR≥95 maintained their statistical significance and a similar magnitude after adjusting for baseline imbalance; the effect of high fidelity clinics on ART initiation after adjusting for baseline imbalance was attenuated.

**Table 3:** Effect of the intervention on PMTCT indicators, stratified by treatment intensity, Tanzania, 2014–2015^a^.

Outcome		Baseline (births in 2014)			Endline (births in 2015)							
	N	Tx^b^ intensity		Control	Tx intensity		Control	DiD^h^ Unadjusted^j^		DiD Adjusted^k^		
		High	Low		High	Low		High	Low	High		Low
								(95% CI^i^)	(95% CI)	(95% CI)		(95% CI)
**Total**	1830	171	391	590	94	210	374	_	_	_		_
**Women retained in care (%)**^**b**^^,^^**c**^	1830	35.0%	35.6%	34.6%	48.6%	42.6%	47.3%	0.9 (-15.5, 17.3)	-5.6 (-19.1, 7.8)	-6.8 (-19.9, 6.3)		-2.0 (-16.0, 11.9)
Full Sample												
**Women retained in care (%)**^**b**^^,^^**c**^	1348	51.5%	45.3%	46.4%	70.2%	61.9%	59.0%	6.1 (-9.1, 21.3)	4.0 (-7.5, 15.5)	-3.6 (-16.4, 9.1)		4.8 (-8.9, 18.4)
Women with evidence of care												
**Women initiating ART**^**d**^	1544	40.6%	50.9%	55.1%	45.9%	44.5%	46.0%	14.4 (4.9, 23.8)***	2.7 (-10.0, 15.3)	8.1 (-2.6, 18.8)		3.8 (-6.1, 13.6)
**Timing of ART initiation**^**e**^	787	27.9	26.7	27.3	23.3	29.3	24.3	-1.6 (-6.5, 3.2)	5.6 (2.8, 8.5)***	-1.5 (-6.1, 3.0)		5.3 (2.4, 8.2)**
**Women with MPR**≥**95%**^**f**^^,^^**g**^	820	18.8%	14.3%	21.2%	34.8%	24.1%	22.8%	14.3 (2.0, 26.6)**	8.2 (-9.2, 25.5)	13.6 (2.5, 24.6)***		4.8 (-14.9, 24.4)
**Women with MPR**≥**80%**^**f**^^,^^**g**^	820	33.2%	24.1%	34.9%	52.5%	41.8%	41.6%	12.6 (-2.4, 27.5)*	11.0 (-6.8, 28.7)	11.6 (-1.6, 24.9)*		5.0 (-11.5, 21.4)

* significant at the α = 0.10 level

**significant at the α = 0.05 level

***significant at the α = 0.01 level

a) estimates were weighted for selection, site size and, at baseline, missing women (see methods)

b) Tx = treatment, grouped into a “high” category, scores above the median, or a “low” category, at or below the median (see methods)

c) Women retained in care calculated for n = 1830 all women in the sample, and n = 1348 women with evidence of HIV care

d) Number of women initiating ART calculated for the n = 1544 women who did not have evidence of beginning ART prior to pregnancy; women were considered to have initiated ART if they had any evidence of ART use between pregnancy and 90 days postpartum

e) Timing of ART initiation calculated for n = 787 women who had an ARV start date on record, and who began ART after pregnancy

f) MPR ≥95 (and MPR≥80) are the women that have 95% adherence (or 80% adherence) or greater according to the MPR calculation, defined as the number of pill days dispensed over the number of days elapsed from the infant’s birth to 90 days postpartum

g) MPR calculated for n = 820 women who had complete ARV dispensing information

h) DiD = difference-in-differences estimate, generated through the linear regression of the outcome with an interaction variable for time and treatment status (see methods)

i) CI = confidence interval

j) Unadjusted for baseline imbalance

k) Adjusted for average number of CHWs per site and percent of HEID testing

## Discussion

This study examined the short-term effectiveness of an intervention utilizing CHWs to strengthen maternal health and PMTCT services in Shinyanga, Tanzania. Our ITT and adjusted analyses revealed that the intervention did not significantly change the primary outcome of retention in care at 90 days postpartum or the secondary outcomes related to Option B+ implementation, such as ART initiation and its timing during the pregnancy. However, both the primary analyses and sensitivity analysis by intervention fidelity suggest that the intervention may have improved postpartum ART adherence, especially among women living in catchment areas where the intervention was implemented with higher intensity. In those sites, we observed a significant increase in the proportion of women achieving at least 95% MPR at 90 days postpartum, an adherence threshold associated with virologic suppression.[22] Although the width of the confidence intervals indicates considerable uncertainty in our estimates, if such an increase in adherence were true, it could have direct benefits on maternal health and secondary benefits on preventing MTCT during breastfeeding.[33]

We hypothesize that the intervention’s effects were primarily manifested on ART adherence among women already enrolled in care and on ART because these women had already wholly or partially overcome economic and/or social obstacles to HIV care, such as transportation costs, lack of disclosure, and/or stigma associated with HIV. For these women, CHWs may have provided a motivating “nudge” to attend scheduled appointments on time, thereby improving the proportion of women with high levels of ART possession (i.e., MPR, which is influenced, by definition, with on-time pharmacy pickups). In contrast, the intervention as designed may have been unlikely to overcome significant barriers for women who were out of care at the start of the intervention or who were forced to disengage from care during their pregnancy. It is well documented, for example, that financial obstacles, lack of HIV disclosure to household members, and stigma can hinder HIV care initiation among pregnant and postpartum women in sub-Saharan Africa.[34–36] The intervention was clearly unable to remove these obstacles, which may explain the lack of a finding on retention in care at 90 days postpartum.

To our knowledge, this is the first rigorous study evaluating the use of CHWs to enhance PMTCT services post-Option B+ rollout in Tanzania. We found that the intervention itself was relatively low cost as well as acceptable to facility staff, CHWs, and patients. The results agree with recent pre-Option B+ research that suggests that utilizing CHWs to implement PMTCT services is feasible,[13] and can potentially aid in virologic suppression.[37] Importantly, CHWs can act as a ‘bridge’ through which women build stronger connections to facility-based services by encouraging them to attend visits by identifying and helping to overcome barriers such as distance by arranging for women’s transportation.[13] This is particularly important in the context of Tanzania’s National Strategy for eMTCT, in which the integration of community involvement in the delivery of community eMTCT and paediatric HIV care and treatment services is a priority.[38] Our findings disagree, however, with data from rigorous studies reporting a connection between CHW delivery of PMTCT services and retention in care.[39–41] This may be due to differences in the interventions themselves and/or because of a lack of statistical power in the current study. In addition, we faced several implementation challenges that may be pertinent in future CHW-based interventions, including heterogeneity in education status of the CHWs as well as a lack of CHW motivation at some sites. The lack of effect on retention in care in this study is particularly important, given the high levels of loss to follow up that have been recently found under Option B+, particularly in the first year postpartum,[8] highlighting anew the need to continue to develop effective interventions that can address this.

In addition to evaluating the intervention’s effectiveness, this study provides evidence of temporal improvements in key outcome indicators among HIV-positive postpartum women. Retention in care, ART initiation and timing of initiation all improved significantly in the control group over time. While there is no way to be certain of the cause of such improvements, it is conceivable that this change is related to the successful adoption of Option B+ and/or other NGO programs that target MTCT. Improvements in these key points of the service cascade are encouraging and worthy of further documentation as Tanzania strives for elimination of MTCT.

This study has several limitations, including most notably imprecise effect estimates due to lack of statistical power. We anticipated *a priori* that, with at least 6 intervention months in each community, we could meet our sample size goal of 39 postpartum women living with HIV infection per facility, as many RCH facilities initiate an average of 11 HIV-infected women on ART per month. In reality, we found that the number of HIV-infected pregnant and postpartum women per site was less than expected (on average, 34 per site), particularly at endline, where the window period for inclusion was only six months and fixed in length by the short period of evaluation mandated by the funding agency. An additional limitation is that we were only able to indirectly monitor the frequency of CHW visits through self-report log forms, which were prone to inaccuracy, due in part to the limited formal education levels of some CHWs. We also collected data retrospectively, which relies on the accuracy on clinic health records, and may therefore be prone to some error. Finally, the difference-in-difference approach assumes equal time trends among treatment and control groups, an assumption we could not verify because of the lack of pre-baseline measures. Nevertheless, we do provide evidence of baseline balance of facility-level variables indicating similarity of the groups at baseline.

This study also has significant strengths. We used a rigorous cluster-randomized design to evaluate short-term intervention effectiveness, contributing high-quality evidence in pragmatic PMTCT research. It also relies on collection of individual-level data linked across numerous medical registers (not aggregate) from study facilities to measure health outcomes. Furthermore, we used a combination intervention implemented by CHWs, which allows for cost-effective and community-sensitive service delivery in the context of workforce shortages.[10,12]

This short-term randomized trial has revealed that, while the intervention was not successful in improving retention in care or ART initiation, it may help to improve adherence. Moreover, facilities that implemented the intervention with a high quality were most successful in improving adherence. This highlights the potential benefits of the program, as well as the importance of high-quality implementation in order to ensure optimal effects. Future iterations of the program may therefore optimize impact by leveraging tools like mobile technology to better track program implementation.[42] Moreover, to more accurately delineate the effect of the intervention, we recommend a redesign of the intervention package that can more actively engage women in the community who have not yet begun vital ART services. By more closely examining both the benefits and challenges of strengthening the CHW cadre, we can work to achieve optimal health outcomes for HIV-positive women pregnant and postpartum women and their children.

## Supporting information

S1 Table Difference-in-difference effect estimates of the community health worker intervention among HIV-positive pregnant and postpartum women in Tanzania using logistic regression models, stratified by treatment group, 2014-2015^a^. (DOCX)

S1 File Impact evaluation protocol. (PDF)

S2 File CONSORT 2010 checklist. (PDF)

S3 File CONSORT extension for cluster trials checklist. (PDF)

S4 File Final dataset. (CSV)

## References

[1] UNAIDS. 2015 Progress Report on the Global Plan Towards the Elimination Of New HIV Infections Among Children and Keeping their Mothers Alive [Internet]. 2015 [cited 6 Sep 2016]. Available: http://www.unaids.org/en/resources/documents/2015/JC2774_2015ProgressReport_GlobalPlan

[2] WHO. World Health Oganization PMTCT Strategic Vision 2010–2015. 2010.

[3] World Health Organization. Consolidated guidelines on the use of antiretroviral drugs for treating and preventing HIV infection: recommendations for a public health approach. 2016; 27466667

[4] United Republic of Tanzania. Global AIDS Response Country Progress Report [Internet]. 2014. Available: http://www.unaids.org/sites/default/files/country/documents/TZA_narrative_report_2014.pdf

[5] World Health Organization. Programmatic Update. Use of Antiretroviral Drugs for Treating Pregnant Women And Preventing HIV Infection in Infants. Geneva; 2012. 26180894

[6] Goga AE, Dinh T-H, Jackson DJ, Lombard CJ, Puren A, Sherman G, et al Population-level effectiveness of PMTCT Option A on early mother-to-child (MTCT) transmission of HIV in South Africa: implications for eliminating MTCT. J Glob Health. Edinburgh University Global Health Society; 2016;6: 20405 doi: 10.7189/jogh.6.020405 27698999 10.7189/jogh.6.020405 PMC5032343

[7] Woldesenbet S, Jackson D, Lombard C, Dinh T-H, Puren A, Sherman G, et al Missed Opportunities along the Prevention of Mother-to-Child Transmission Services Cascade in South Africa: Uptake, Determinants, and Attributable Risk (the SAPMTCTE). PloS One. Public Library of Science; 2015;10: e0132425 doi: 10.1371/journal.pone.0132425 26147598 10.1371/journal.pone.0132425 PMC4492960

[8] Haas AD, Tenthani L, Msukwa MT, Tal K, Jahn A, Gadabu OJ, et al Retention in care during the first 3 years of antiretroviral therapy for women in Malawi’s option B+ programme: an observational cohort study. Lancet HIV. 2016;3: e175–e182. doi: 10.1016/S2352-3018(16)00008-4 27036993 10.1016/S2352-3018(16)00008-4 PMC4904064

[9] Geldsetzer P, Yapa HMN, Vaikath M, Ogbuoji O, Fox MP, Essajee SM, et al A systematic review of interventions to improve postpartum retention of women in PMTCT and ART care. J Int AIDS Soc. 2016;19. 10.7448/IAS.19.1.20679 PMC4846797 27118443

[10] Kwesigabo G, Mwangu MA, Kakoko DC, Warriner I, Mkony CA, Killewo J, et al Tanzania’s health system and workforce crisis. J Public Health Policy. 2012;33 Suppl 1: S35–44. doi: 10.1057/jphp.2012.55 23254848 10.1057/jphp.2012.55

[11] World Health Organization. The World Health Report 2006: Working Together for Health. Geneva; 2006.

[12] Anyangwe SC, Mtonga C. Inequities in the global health workforce: the greatest impediment to health in sub-Saharan Africa. Int J Env Res Public Health. 2007;4: 93–100. 17617671 10.3390/ijerph2007040002 PMC3728573

[13] Mwai GW, Mburu G, Torpey K, Frost P, Ford N, Seeley J. Role and outcomes of community health workers in HIV care in sub-Saharan Africa: a systematic review. J Int AIDS Soc. 2013;16: 18586 doi: 10.7448/IAS.16.1.18586 24029015 10.7448/IAS.16.1.18586 PMC3772323

[14] Mubita, B. Engaging communities to accelerate children’s access to HIV prevention, treatment care and support services. 21st International AIDS Conference; 2016 Jul 18; Durban, South Africa.

[15] Beeson, L. Acceptability of integrated home-based screening for HIV, TB and non-communicable diseases in rural South Africa. 21st International AIDS Conference; 2016 Jul 18; Durban, South Africa.

[16] World Health Organization Global Health Observatory (GHO). Density of physicians (total number per 1000 population, latest available year) [Internet]. 21 Aug 2014.

[17] UNICEF Health Section, Program Division. Access to healthcare through comunity health workers in East and Southern Africa. New York: UNICEF; 2014.

[18] McCoy SI. Short-term effectiveness of a community health worker intervention for HIV-infected pregnant women in Tanzania to improve treatment adherence and retention in care. 21st International AIDS Conference; 2016 Jul 17; Durban, South Africa. 10.1371/journal.pone.0181919 PMC5578486 28859083

[19] National Bureau of Statistics (NBS) [Tanzania] and ICF Macro. Tanzania Demographic and Health Survey 2010. Dar es Salaam, Tanzania: NBS and ICF Macro; 2011.

[20] StataCorp. STATA Statistical Software: Release 13. College Station, TX; 2013.

[21] Webb, K. Implementation Research in Action: Increasing Facility-based Delivery and Reducing HIV-related Complications in Mashonaland Central, Zimbabwe [Internet]. 20th International AIDS Conference 2014; 2014 Jul 20. Available: http://www.ophid.co.zw/downloads/Webb_AIDS2014_Action%20Research%20paper%20poster_14_07_14_FINAL.pdf

[22] McMahon JH, Jordan MR, Kelley K, Bertagnolio S, Hong SY, Wanke CA, et al Pharmacy adherence measures to assess adherence to antiretroviral therapy: review of the literature and implications for treatment monitoring. Clin Infect Dis Off Publ Infect Dis Soc Am. 2011;52: 493–506. doi: 10.1093/cid/ciq167 21245156 10.1093/cid/ciq167 PMC3060901

[23] Messou E, Chaix ML, Gabillard D, Minga A, Losina E, Yapo V, et al Association between medication possession ratio, virologic failure and drug resistance in HIV-1-infected adults on antiretroviral therapy in Cote d’Ivoire. J Acquir Immune Defic Syndr. 2011;56: 356–64. doi: 10.1097/QAI.0b013e3182084b5a 21191309 10.1097/QAI.0b013e3182084b5a PMC3050083

[24] Goldman JD, Cantrell RA, Mulenga LB, Tambatamba BC, Reid SE, Levy JW, et al Simple adherence assessments to predict virologic failure among HIV-infected adults with discordant immunologic and clinical responses to antiretroviral therapy. AIDS Res Hum Retroviruses. 2008;24: 1031–5. doi: 10.1089/aid.2008.0035 18724803 10.1089/aid.2008.0035 PMC2747786

[25] Hong S, Nachega J, Jerger L, Cohen S, Jonas A, Badi A, et al. Medication Possession Ratio Predictive of Short-term Virologic and Immunologic Response in Individuals Initiating ART: Namibia. 19th Conference on Retroviruses and Opportunistic Infections; 2012; Seattle.

[26] Bangsberg DR. Less than 95% adherence to nonnucleoside reverse-transcriptase inhibitor therapy can lead to viral suppression. Clin Infect Dis Off Publ Infect Dis Soc Am. 2006;43: 939–41. doi: 10.1086/507526 16941380 10.1086/507526

[27] Gertler PJ, Martinez S, Premand P, Rawlings LB, Vermeersch CM. Impact Evaluation in Practice. Washington, D.C.: World Bank; 2010.

[28] Qualtrics. Qualtrics Survey Software [Internet]. Provo, Utah, USA; 2015. Available: http://www.qualtrics.com

[29] Hellevik O. Linear versus logistic regression when the dependent variable is a dichotomy. Qual Quant. 2009;43: 59–74.

[30] StataCorp. STATA manual: svy command [Internet]. College Station, TX: Stata Press; Available: http://www.stata.com/manuals13/svy.pdf

[31] Andersen C, Njau PF, McCoy SI. Loss to Follow-up Among Option B+ Patients in Shinyanga Region. Unpublished.; 2014.

[32] StataCorp. STATA manual: loneway command [Internet]. College Station, TX: Stata Press; Available: http://www.stata.com/manuals13/rloneway.pdf

[33] Slater M, Stringer EM, Stringer JS. Breastfeeding in HIV-positive women: What can be recommended? Paediatr Drugs. 2010;12: 1–9. doi: 10.2165/11316130-000000000-00000 20034337 10.2165/11316130-000000000-00000

[34] Turan JM, Nyblade L. HIV-related stigma as a barrier to achievement of global PMTCT and maternal health goals: a review of the evidence. AIDS Behav. 2013;17: 2528–39. doi: 10.1007/s10461-013-0446-8 23474643 10.1007/s10461-013-0446-8

[35] Medley A, Garcia-Moreno C, McGill S, Maman S. Rates, barriers and outcomes of HIV serostatus disclosure among women in developing countries: implications for prevention of mother-to-child transmission programmes. Bull World Health Organ. 2004;82: 299–307. 15259260 PMC2585956

[36] Duff P, Kipp W, Wild TC, Rubaale T, Okech-Ojony J. Barriers to accessing highly active antiretroviral therapy by HIV-positive women attending an antenatal clinic in a regional hospital in western Uganda. J Int AIDS Soc. 2010;13: 37 doi: 10.1186/1758-2652-13-37 20863399 10.1186/1758-2652-13-37 PMC2954932

[37] Jaffar S, Amuron B, Foster S, Birungi J, Levin J, Namara G, et al Rates of virological failure in patients treated in a home-based versus a facility-based HIV-care model in Jinja, southeast Uganda: a cluster-randomised equivalence trial. Lancet. 2009;374: 2080–9. doi: 10.1016/S0140-6736(09)61674-3 19939445 10.1016/S0140-6736(09)61674-3 PMC2806484

[38] MoHSW. Tanzania elimination of mother to child transmission of HIV plan, 2012–2015. Tanzan Elimin Mother Child Transm HIV Plan 2012–2015. 2012; 108.

[39] Chang LW, Kagaayi J, Nakigozi G, Ssempijja V, Packer AH, Serwadda D, et al Effect of peer health workers on AIDS care in Rakai, Uganda: a cluster-randomized trial. PloS One. 2010;5: e10923 doi: 10.1371/journal.pone.0010923 20532194 10.1371/journal.pone.0010923 PMC2880005

[40] Torpey KE, Kabaso ME, Mutale LN, Kamanga MK, Mwango AJ, Simpungwe J, et al Adherence support workers: a way to address human resource constraints in antiretroviral treatment programs in the public health setting in Zambia. PloS One. 2008;3: e2204 doi: 10.1371/journal.pone.0002204 18493615 10.1371/journal.pone.0002204 PMC2377331

[41] Igumbor JO, Scheepers E, Ebrahim R, Jason A, Grimwood A. An evaluation of the impact of a community-based adherence support programme on ART outcomes in selected government HIV treatment sites in South Africa. AIDS Care. 2011;23: 231–6. doi: 10.1080/09540121.2010.498909 21259136 10.1080/09540121.2010.498909

[42] Thurston, B. Effectiveness of an electronic dashboard for supervising and monitoring community health workers (CHWs) to scale up voluntary medical male circumcision (VMMC) in Zambia [Internet]. 21st International AIDS Conference; Durban, South Africa. Available: 21st International AIDS Conference

