# Supplementary material for: Short-term effectiveness of a community health worker intervention for HIV-infected pregnant women in Tanzania to improve treatment adherence and retention in care: A cluster-randomized trial
Source: PLoS One. 2017 Aug 31;12(8):e0181919. doi: 10.1371/journal.pone.0181919 (PMC5578486; doi:10.1371/journal.pone.0181919)
Supplement: S1 Table — (DOCX) [file pone.0181919.s001.docx]

**S1 Table. Difference-in-difference effect estimates of the community health worker intervention among HIV-positive pregnant and postpartum women in Tanzania using logistic regression models, stratified by treatment group, 2014-2015**

|  |  | **Control Group** | | **Treatment Group** | |  | |  |  | |
| --- | --- | --- | --- | --- | --- | --- | --- | --- | --- | --- |
|  | **N** | **Baseline**  Log[Y]^f^(SE)^g^ | **Endline**  Log[Y] (SE) | **Baseline**  Log[Y] (SE) | **Endline**  Log[Y] (SE) | **DiD^h^  Unadjusted**  **β (95% CI^j^)** | **DiD Adjusted^i^**  **β (95% CI^j^)** | | |  |
| **Women retained in care (%)^b^** | 1830 | -0.64 (0.11) | -0.11 (0.22) | -0.61 (0.08) | -0.19 (0.17) | -0.11 (-0.66, 0.44) | -0.19 (-0.89, 0.50) | | |  |
| *Full Sample* |  |  |  |  |  |  |  |  |  |  |
| **Women retained in care (%)^b^** | 1348 | -0.14 (0.11) | 0.36 (0.11) | -0.08 (0.12) | 0.64 (0.14) | 0.22 (-0.24, 0.67) | 0.11(-0.50, 0.73) | | |  |
| *Women with evidence of care* |  |  |  |  |  |  |  |  |  |  |
| **Women initiating ART^c^** | 1544 | 0.21 (0.13) | -0.16 (0.21) | -0.15 (0.13) | -0.20 (0.15) | 0.32 (-0.12, 0.65) | 0.18 (-0.35, 0.71) | | |  |
| **Women with MPR≥95%(%)^d,e^** | 820 | -1.31 (0.18) | -1.22 (0.18) | -1.65 (0.28) | -0.89 (0.25) | 0.66 (-0.03, 1.35)* | 0.55 (-0.17, 1.27) | | |  |
| **Women with MPR≥80% (%) ^d,e^** | 820 | -0.62 (0.19) | -0.34 (0.17) | -0.95 (0.20) | -0.13 (0.23) | 0.54 (-0.02,1.10)* | 0.40 (-0.14, 0.93) | | |  |

* significant at the α = 0.10 level ** significant at the α = 0.10 level

1. Estimates were weighted for selection, site size and, at baseline, missing women
2. Women retained 0-90 days postpartum, calculated for n=1830 all women in the sample, and n=1348 women with evidence of HIV care
3. Number of women initiating ART calculated for the n=1544 women who did not have evidence of beginning ART prior to pregnancy; women were considered to have initiated ART if they had any evidence of ART use between pregnancy and 90 days postpartum
4. MPR ≥95 (and MPR≥80) are the women that have 95% adherence (or 80% adherence) or greater according to the medicine possession ratio (MPR) calculation, defined as the number of pill days dispensed over the number of days elapsed from the infant’s birth to 90 days postpartum
5. MPR calculated for n=820 women who had complete ARV dispensing information
6. Log[Y]= estimate of log-odds of outcome among each group (baseline treatment, endline treatment, etc.)
7. SE = standard error
8. DiD = difference-in-differences estimate, unexponentiated coefficient (β, log odds) of the interaction term for time and treatment status, generated through the logistic regression (see methods)
9. Adjusted model additionally adjusted for factors imbalanced at baseline (HEID testing and number of CHWs at the facility)
10. CI = confidence interval
