# Supplementary material for: Short-term effectiveness of a community health worker intervention for HIV-infected pregnant women in Tanzania to improve treatment adherence and retention in care: A cluster-randomized trial
Source: PLoS One. 2017 Aug 31;12(8):e0181919. doi: 10.1371/journal.pone.0181919 (PMC5578486; doi:10.1371/journal.pone.0181919)
Supplement: S1 File — (PDF) [file pone.0181919.s002.pdf]

**IMPACT EVALUATION PROTOCOL**

# Integration of Community Maternal, Newborn, and Child Health (MNCH) Services with Prevention of Mother-to-Child HIV Transmission (PMTCT) using Community Health Workers to Enhance Retention in Care and Improve Adherence to Antiretroviral Therapy in Tanzania


**Impact Evaluation Team:**

**Amref Health Africa, Tanzania**

Rita Noronha
Deputy Country Director

David Ngilangwa

Awene Gavylole

**University of California, Berkeley**

Sandra McCoy, MPH, PhD
Assistant Professor of Epidemiology

Nerissa Nance
MPH Candidate, Epidemiology/Biostatistics

**Tanzania Ministry of Health and Social Welfare**

Prosper Njau, MD MPH
Programme Officer, PMTCT

NtuliKapologwe, MD MPH
Shinyanga Regional Medical Officer


## 1. SUMMARY


Amref Health Africa Tanzania is implementing a pilot program in Shinyanga Region, Tanzania to integrate maternal, newborn and child health (MNCH) and prevention of mother to child transmission (PMTCT) services using community health workers (CHWs). The foundation of the multi-pronged intervention is linkage of CHWs to health facility staff for mentorship, supportive supervision, and coordination of tasks. In addition, CHWs will distribute the "Action Birth Card", an interactive planning tool that links MNCH services delivered at the community level, such as breastfeeding counseling, to PMTCT and human immunodeficiency virus (HIV) treatment and care services delivered at the facility. The card will be targeted to both HIV-infected and uninfected pregnant women and asks them to define and overcome barriers to reproductive health services related to PMTCT and WHO's core health indicators.[1] Furthermore, CHWs in the intervention will provide monthly adherence counseling to women on antriretroviral therapy (ART) and will coordinate with health facilities to trace HIV-infected pregnant women who are lost to follow-up. Together, these strategies will newly integrate community-based MNCH services with HIV prevention, treatment, and care, bridging the gap between community and facility, and enhancing the potential benefits of Option B+.

Using a cluster randomized design, we will evaluate whether this approach: 1) results in earlier initiation of ART; 2) increases the proportion of HIV-infected pregnant women who are retained in care; and 3) increases adherence. We will also examine whether there is impact heterogeneity depending on women's intensity of exposure to the intervention, and we will determine whether the intervention is acceptable and feasible for CHW, health facility staff, and pregnant and postpartum women. The results will be germane to local and regional authorities,

the Ministry of Health and Social Welfare, community organizations, and donors about whether this strategy can improve outcomes in the HIV care continuum.

## 2. PROBLEM STATEMENT

Globally, the number of children born with HIV-infection declined 35% during 2009-2012, a reduction partly attributable to antiretroviral (ARV) prophylaxis preventing 670,000 children from acquiring HIV infection in low- and middle-income countries during this period.[2] However, despite this substantial achievement in PMTCT, there are still significant challenges facing the global effort to achieve UNAIDS' goal of "virtual elimination" of mother-to-child transmission (eMTCT) by 2015.[3] To prevent transmission to their infants, HIV-positive pregnant women need to receive a series of services including antenatal care (ANC), HIV testing, disease staging, and ART or ARV prophylaxis. In addition, HIV-exposed infants should receive ARV prophylaxis, be tested for HIV infection, be exclusively breastfed for the first 6 months of life, and receive cotrimoxazole prophylaxis to prevent opportunistic infections.[4] Although these services have been shown to reduce the risk of MTCT to <5% in breastfeeding populations and <2% in non-breastfeeding populations, in sub-Saharan Africa, 49% of HIV-infected pregnant women are lost between ANC registration and delivery and miss some or all of the essential PMTCT services.[4-6] Consequently, only 57–70% percent of pregnant women living with HIV receive ART or ARV prophylaxis, one of the key steps of the cascade.[2]

Increasing retention in the PMTCT cascade is particularly critical in high-burden, priority countries like Tanzania, where 6.2% of women aged 15-49 are HIV-positive[7] and nearly one quarter of pregnant women living with HIV do not receive ARVs/ART for PMTCT.[2] Notably, in Tanzania, achievements in PMTCT could have important impacts on the national epidemic, as 1 out of every 5 new HIV infections are due to mother-to-child transmission – an estimated 36,225 infections per year.[8] In September 2013, Tanzania began rolling out 'Option B+' to all 6,270 reproductive and child health (RCH) facilities, the PMTCT strategy whereby HIV-infected pregnant women receive ART throughout their lives regardless of clinical stage.[9,10] By simplifying service delivery, drug regimen, and reducing the number of steps that mothers need to negotiate (such as CD4 testing), Option B+ is expected to improve retention in the PMTCT cascade.[10] However, the postulated benefits of Option B+ are predicated on mothers remaining on lifelong ART, starting at the time of HIV diagnosis. Thus, a critical unresolved issue is whether *asymptomatic* women who learn their HIV status during pregnancy will initiate and adhere to ART under Option B+.

## 3. BACKGROUND AND REVIEW OF LITERATURE

*PMTCT & Option B+*

Malawi was the first to implement the Option B+ strategy, and the initial monitoring and evaluation data were encouraging: Option B+ dramatically increased ART coverage among HIV-infected pregnant women, among whom 77% continued to receive treatment at 12 months, comparable to 80% of adults in the national program.[10] However, more recent cohort data paint a decidedly less optimistic picture, as Option B+ patients who started ART during pregnancy were five times more likely to never return after their initial clinic visit compared to women who started ART because they were treatment eligible.[11] In the same analysis, pregnant women who started ART on the day of HIV diagnosis had the highest loss to follow-up rates.[11] Furthermore, a meta-analysis of 51 studies, including over 20,000 HIV-infected pregnant and postpartum women, found that adherence (80% or higher) was a modest 53% in the postpartum period compared to 76% antepartum.[12] <u>Together, these data highlight the central challenge for eMTCT</u>

and keeping women alive: preventing attrition from the treatment and care cascade and achieving high levels of adherence to ART.[3,13,14]

*Evidence Supporting Community-Based MNCH & PMTCT Integration*

Effectiveness of Facility-Based MNCH and PMTCT Integration: The integration of *facility-based* MNCH and PMTCT services is recommended by WHO and PEPFAR.[4,15-17] A systematic review found that ART integration into MNCH facilities significantly improves ART uptake (RR: 1.37, 95% CI: 1.05–1.79).[15] However, only one study has evaluated the effect of facility-based integration of MNCH and PMTCT on retention in care[18] and no studies have evaluated ART adherence.

*Effectiveness of CHWs for HIV-Related Outcomes*

In the HIV/AIDS realm, rigorous data on the effectiveness of integrating CHWs into HIV/AIDS treatment and care services are scarce. Although CHWs have been used to perform a variety of HIV/AIDS related activities including HIV testing, behavior change counseling, adherence support, and delivery of ART, a 2013 systematic review of the effectiveness of integrating CHWs with HIV care in sub-Saharan Africa found a weak evidence base. Of the 21 studies identified, five were qualitative, only 3 were randomized trials, and the majority of observational studies lacked control groups.[19] Nevertheless, the findings from several higher-quality studies suggest the potential of integrating CHWs with HIV treatment and care. A cluster randomized trial in Uganda found that a community-based peer health worker intervention cut loss to follow-up rates at 24 months in half, from 4.4% to 2.1%, and decreased virologic failure rates ≥96 weeks into ART.[20] Positive benefits on retention in care were also reported in quasi-experimental studies of CHW in Zambia,[21] South Africa,[22] and Malawi.[23] In the South African study, ART patients with CHW adherence support had higher treatment pickup rates (95% vs. 67%, $p=0.02$) and were more likely to have a suppressed viral load after 6 months of treatment (70% vs. 30%, $p<0.01$).[22] In addition, a home-based HIV-care strategy in Uganda using lay health workers was found to be as effective as a clinic-based strategy on viral suppression after 6 months of ART.[24] The benefits observed in these studies may be because CHWs can help to demystify HIV, counteract stigma, and "bridge" the community and health facility.[19]

*Effectiveness of CHWs for PMTCT Outcomes*

In the proposed study, we hypothesize that the new integration of community-based MNCH services with PMTCT using CHWs will increase demand for ART among HIV-infected pregnant and breastfeeding women and will improve retention in care and adherence to ART. This hypothesis is bolstered by a small but growing body of evidence that such a strategy can be effective and also help to mitigate the health workforce crisis.[25] In South Africa, mothers in the *Philani* program who received antenatal and postnatal home visits by CHWs were more likely to administer infant nevirapine at birth, correctly treat infants with zidovudine, and practice one feeding method for the first 6 months of the infants life.[26] Overall, the odds of completing all services in the PMTCT cascade was nearly twice as high in the CHW intervention group compared to standard care.[26] Also in South Africa, the *Goodstart* study found that CHW visits to pregnant and postpartum women increased exclusive breastfeeding among HIV-infected women and increased infant weight and length-for-age z-scores, although no differences were found for MTCT or HIV-free survival.[27] Similar increases in PMTCT service utilization were observed in Malawi's *Tingathe* PMTCT programme, a CHW-based patient case management intervention.[28] Although these studies are promising, these studies have a clear focus on preventing infant infections and improving infant health; none evaluated whether the mother was retained in care after her baby was delivered and whether she was adherent to ART. Furthermore, none have been conducted since the implementation of Option B+, which is a new and integrative approach to PMTCT with specific challenges for health care delivery.

In summary, the integration of ART services with maternal, newborn, and child health services (MNCH) is essential for PMTCT and has been demonstrated to improve ART coverage among HIV-infected pregnant and postpartum women.[4,15,17] However, despite the proven effectiveness of delivering MNCH and other health services at the community level,[29] there is a paucity of evidence on the effect of integrating MNCH and PMTCT services using CHWs. CHWs are integral members of the health system who have received training to promote health or to carry out some healthcare services, but who are not healthcare professionals.[19,29] It is well known that CHWs can enhance the reach, coverage, and quality of HIV services delivered to the general population,[19] but specific evidence for leveraging this essential cadre to link community-based MNCH services with the PMTCT cascade is small and primarily focused on infant outcomes.[15,26-28] Integration of community-based MNCH services with PMTCT is especially critical because Tanzania is scaling up 'Option B+', a PMTCT strategy whereby HIV-infected pregnant and breastfeeding women are immediately initiated on lifelong ART after diagnosis. Given the significant potential for loss to follow-up after ART initiation, coupled with the high workload for health care workers, there is a strong argument for integrating community-based MNCH activities with PMTCT services using CHWs, especially adherence counseling and tracing pregnant women who never sought care or who fell out of care.

## 4. STUDY SETTING AND RATIONALE

Our study will be conducted in Shinyanga, one of Tanzania's 30 regions in the northern "Lake Zone" where HIV prevalence is 7.4% (8.1% among women)[7]. Under Tanzania's Reproductive Child Health Platform Approach and Option B+, ANC, postnatal care, and HIV treatment and care are now provided in the majority of Tanzania's 6,270 reproductive and child health (RCH) clinics.[30] There are approximately 12,000 CHWs in Tanzania,[31] including 1,512 CHWs in Shinyanga Region who serve 490 villages. Under the current structure, however, CHWs in Shinyanga do not participate in any activities related to the provision of HIV care for HIV-infected pregnant women, PMTCT, or Option B+.

The current study would connect these two separate programs and subsequently evaluate the effectiveness of providing Option B+ through CHW care. While previous studies have found CHWs to be effective,[19]they have a clear focus on preventing infant infections and improving infant health; none evaluated whether the mother was retained in care after her baby was delivered and whether she was adherent to ART. Furthermore, none have been conducted since the implementation of Option B+, which is a new and integrative approach to PMTCT with specific challenges for health care delivery.

## 5. DESCRIPTION OF THE INTERVENTION

***Mama na Mtoto Pamoja* (Mother and Child Together)**

The intervention, implemented by our partner Amref Health Africa Tanzania, will integrate community-based MNCH and PMTCT services by implementing the following policies, activities, and tools in 15 communities:

1. <u>Formal Linkage of CHW to RCH clinics:</u> As noted above, although an informal relationship exists between CHWs and a single health facility for referral purposes in relation to maternal and child health services, there is currently no process for CHWs to review activities with clinic staff, receive mentorship, and determine which women have been lost to follow-up. We will assign each CHW to a nurse in the RCH clinic for a monthly meeting to discuss their activities. This will overcome the limitations of the current model whereby CHW are not engaged with health facility staff for supervision and supportive mentorship.

2. Action Birth Card: The “Action Birth Card” is an interactive, integrated health planning tool distributed by CHWs that links MNCH services delivered at the community level to PMTCT and HIV treatment and care services delivered at the facility. It asks pregnant and breastfeeding women to define and overcome barriers to reproductive health services related to PMTCT and WHO’s core health indicators for women and children’s health.[1] Furthermore, the card facilitates discussion between CHWs and their clients and serves as a user-friendly record of completed services and behaviors (e.g., breastfeeding) for maternal health, PMTCT, HIV treatment, family planning, and infant health.

   The Action Birth Card will be distributed by CHWs at their first meeting with pregnant and postpartum women, discussed at all subsequent monthly meetings, and collected after the infant has been tested for HIV infection, 6-8 weeks postpartum. The card, containing health content relevant to *all* women, will be targeted to both HIV-infected and uninfected women to prevent stigma.

3. Tracing of treatment defaulters: At their monthly meetings with RCH staff, CHWs will review the ART register to identify women who missed scheduled appointments and those who are lost to follow-up according to the local definition: non-attendance at three successive scheduled appointments with two unsuccessful follow-up attempts. CHWs will attempt to contact these women via mobile phone or home visit and re-engage them in care. CHWs will provide updates to the clinic about their progress and will coordinate tracing activities with those of the clinic (if any).

4. Adherence counseling for HIV-infected women on ART: CHWs will use the existing adherence counseling manual used by community-based home based care volunteers for people living with HIV, with additional messaging customized for asymptomatic women (as many recently diagnosed pregnant women will be asymptomatic). Adherence counseling will be conducted during the monthly CHW home visits.

The intervention’s theory of change is shown below in ***Figure 1***, including the process and impact indicators that will be measured as part of the impact evaluation. Currently, CHWs in Shinyanga Region (and elsewhere in Tanzania) refer pregnant women to ANC in addition to other health promotion activities. Once registered for ANC, pregnant women may or may not complete the services in the PMTCT cascade, including starting and adhering to ART (if HIV-infected), and they may disengage and re-engage in care. In the current study, we will implement an intervention to integrate community-based MNCH services and PMTCT using CHWs. We hypothesize that this approach will result in HIV-infected pregnant and postpartum women initiating ART earlier as well as better retention in care and improved adherence.

## 6. DESCRIPTION OF THE EVALUATION

### *6.1. Evaluation Questions*

In tandem with the program, we will conduct an impact evaluation to measure the effectiveness of the program. The primary objectives of the evaluation will be to answer the following questions:

1. What is the impact of integrating community-based MNCH and PMTCT services using CHWs in Shinyanga, Tanzania on:
   a. The timing of ART initiation among HIV-infected pregnant and postpartum women;

b. Retention of HIV-infected women in care 90 days postpartum; and
c. Adherence to ART at 90 days postpartum.

2. Is the impact of integrating community-based MNCH and PMTCT services on retention in care and adherence greater among women who had more contact with CHWs?

3. Is integration of community-based MNCH and PMTCT services acceptable and feasible for CHW, health facility staff, and pregnant and postpartum women, and are there potential spillover benefits on other maternal health outcomes?

**FIGURE 1. Theory of Change.** Integration of community health workers (CHWs) into the prevention of mother-to-child HIV transmission (PMTCT) cascade at RCH clinics, with associated process and impact indicators. Boxes within the grey arrow show the steps of the PMTCT cascade for HIV-infected pregnant women, including lifelong antiretroviral therapy. The dashed line and circles outside the grey arrow show the process of defaulting and re-engaging in care. CHW will support HIV-specific services and re-engagement in care through the Action Birth Card (bold arrows). Adapted from Kranzer (2012) and Bhardwajl (2014). ANC=antenatal care; ART=antiretroviral therapy; EID=early infant diagnosis.

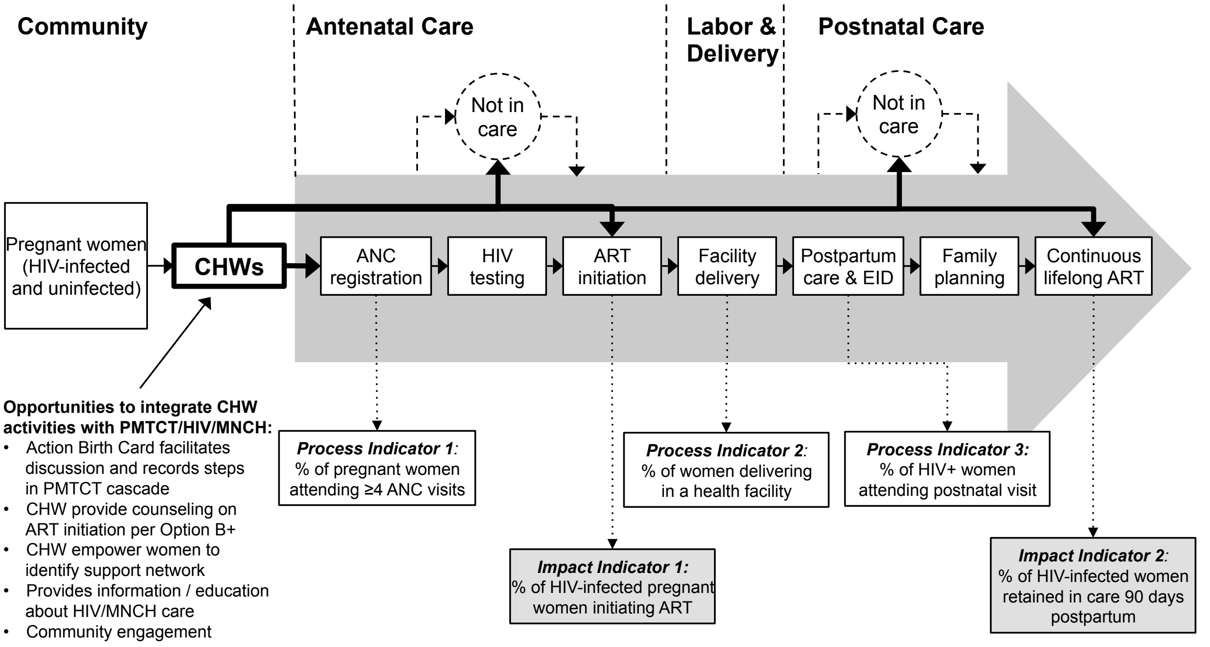


## *6. 2 Impact and Process Indicators*

Impact Outcomes:

1. Timing of ART initiation: The timing of ART initiation among HIV-infected pregnant women according to gestational age (The number weeks since the first day of the last menstrual period (LMP)).We hypothesize that women in intervention communities will initiate ART earlier than women in comparison communities.

2. Retention in care: Whether an HIV-infected pregnant or postpartum woman on ART is still in care 90 days postpartum (binary 1/0). In Tanzania, postpartum women on ART are typically scheduled for clinical monitoring for their own health at 7, 28, and 42 days (6

week postnatal visit) and then every 30 days thereafter until transfer to the HIV clinic at 18 months. We define retention as a woman who attended her scheduled visit between 60-90 days postpartum.

3. Adherence: We will measure adherence with the medication possession ratio (MPR), the proportion of days when ART was prescribed and an individual is in possession of ART (determined with pharmacy dispensing data).[32] MPR is associated with short-term virologic outcomes.[32-35,24]We will determine the proportion of women with MPR ≥95% at 90 days postpartum (binary 1/0).

Process Outcomes:

In addition to the impact outcomes, we have identified three process outcomes to ensure that we assess potential spillover benefits on other maternal and child health outcomes. Thus, as part of Evaluation Question #3, we will also collect data on the following three process outcomes in addition to acceptability and feasibility:

1. ≥4 ANC visits: the proportion of pregnant women attending the WHO recommended ≥4 ANC visits;[36]

2. Facility delivery: the proportion of pregnant women who deliver in a health facility; and

3. Postpartum visit: the proportion of HIV-infected women who attend the 6-week postpartum visit.

4. Early infant HIV diagnosis (EID):the proportion of HIV-exposed infants who receive an HIV test at 6-8 weeks.

*6.3 Evaluation Design*

The impact evaluation will evaluate the effect of integrating community-based MNCH and PMTCT services using CHWs in Shinyanga, Tanzania. Using a cluster randomized design, we will allocate 30 villages to the treatment or comparison condition and measure the community-level outcomes at baseline (0 months) and endline (9 months) using routinely collected data from CHWs and health facilities. We will determine the impact of the intervention on the primary outcomes of ART initiation, retention in care, and adherence to ART (**Evaluation Question #1**). We will determine whether the effect of the intervention is stronger among women who received more visits from CHWs(**Evaluation Question #2**). Lastly, we will conduct a mixed methods process evaluation with the primary stakeholders, including but not limited to CHWs, clinic staff, and pregnant and postpartum women, to assess the intervention's: 1) acceptability; 2) feasibility; and 3) potential spillover benefits on other outcomes (**Evaluation Question #3**). If the data from the evaluation supports scale-up of the integrated strategy, we will use these data to refine the intervention protocols for wider adoption within Tanzania and elsewhere.

*6.4 Sample Size and Power*

In health facilities where there are <50 HIV-infected pregnant or postpartum women in the baseline or endline cohorts (determined with the ANC register), we will abstract data from the ART register on all women. At health facilities where there are ≥50 HIV-infected pregnant or postpartum women in the cohorts, we will abstract a predetermined sampling fraction such that 75% of women are sampled from cohorts with 50-100 women, 50% of women are sampled from cohorts with 100-200 women, etc. This approach will ensure that the sample size is adequate to detect a minimum increase in retention in care at 90 days postpartum.

We have powered the study for the outcome of retention in care 90 days postpartum. In our aforementioned preliminary analysis of pregnant women in Shinyanga, we found that more than

36% of HIV-infected pregnant women who initiated ART were lost within 100 days.[37] Thus, we will conservatively assume that in the comparison communities, 67% of women will be retained in care at 90 days postpartum. For logistical and budgetary reasons, especially given the rapid nature of the impact evaluation, we have determined that we can reasonably study 15 intervention communities (a total of 30 CHW, 2 per village, and 15 RCH facilities) and 15 comparison communities.

If we assume an ICC of 0.05 and that the intervention will increase the proportion of women who are retained in care 90 days postpartum from 67% to 80% (detectable effect size of 13 percentage points, or 20%), we will require at least 36 observations per community, or 537 observations from the intervention and comparison groups, to have 80% power to reject the null hypothesis of no effect. Obtaining data from at least 36 HIV-infected women per facility is well within the normal volume of clients at RCH clinics, as many RCH clinics initiate an average of 11 HIV-infected women on ART/ARVs per month. With at least 6 intervention months in each community, we will ensure that we can easily meet this goal.

*6.5 Randomization Procedures*

We will identify 30 similarly sized communities in Shinyanga District Council. For potential inclusion, communities must have an active cadre of CHWs, have similar numbers of households and schools, be noncontiguous or separated by natural boundaries, and be within 5 km of a RCH facility that is providing Option B+. The impact evaluation team will assign communities to the intervention or comparison group using simple randomization (using the sample command in STATA), consistent with previous studies.[26]

## 7. METHODOLOGY

*7.1 Definition of Baseline and Endline Cohorts of HIV-Infected Pregnant Women*

We will retrospectively abstract data from routinely collected information in CHW logs and facility registries of patient medical information (see ***Figure 1*** for an overview of the impact and process outcomes). The research team will collect data from two 6-month cohorts (baseline and endline) of women who are 90 days postpartum to ensure that we can measure retention in care and adherence at 90 days postpartum (***Figure 4***):

- The baseline cohort will include HIV-infected women who were 90 days postpartum between July 1 and December 31, 2014; this corresponds to infants born between April 1 and September 30, 2014. These women are unexposed to the intervention.
- The endline cohort will include HIV-infected women who are 90 days postpartum between May 1 and October 31, 2015, corresponding to infants born between February 1 and July 31, 2015. These women will have been exposed to the intervention during the last 4-9 months of their pregnancy and during their entire 0-90 day postpartum period. This is a reasonable amount of exposure to the intervention given that, on average, women in Tanzania are 5.4 months pregnant at their first ANC visit when they have the initial opportunity to initiate ART.[38]

**FIGURE 4.** Cohorts for Baseline and Endline Data Collection

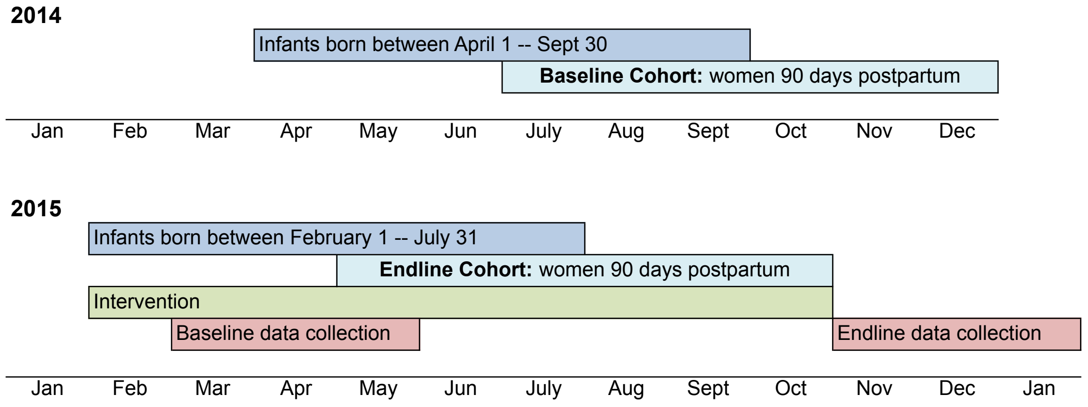


## *7.2. Data Sources*

Data will be abstracted from the following sources, using the woman's clinic identification number (clinic ID) as the unique code to link across data sources:

- <u>CHW logs:</u> CHWs maintain standardized logs of pregnant and postpartum women in their catchment area. At the start of the study, we will modify the log form to include women's clinic ID. We will retrospectively abstract the clinic ID along with demographic data about both HIV-infected and uninfected women in the baseline or endline cohorts, as CHW in comparison villages may not know women's HIV serostatus. Note that this is a significant strength of our study, as we will have a community-based measure of pregnant women, including women who do not utilize RCH services.
- <u>ANC register:</u> Contains information about all women who booked for ANC, including number of visits, HIV testing results, health facility delivery, and attendance at the postnatal visit. We will abstract these data for each woman in the baseline or endline cohort and match their health information with CHW log information.
- <u>ART register:</u> Contains information about HIV-infected pregnant or postpartum women who are initiated on ART at RCH clinics. For all HIV-infected women in the baseline or endline cohorts, we will match them to the ART register to determine whether they initiated ART and attended visits.
- <u>CTC2 Cards:</u> Contains individual medical record data for HIV-infected women. We will abstract data from these cards as needed, to supplement data in the ART register.
- <u>Pharmacy logbook:</u> Contains ART pick-up dates and quantity for HIV-infected women.

**Table 1.**

| Evaluation Question | Data Sources | Outcome / Indicator(s) |
|---|---|---|
| 1. Impact of the intervention on: | | |
| a. timing of ART initiation | ART register<br>CTC2 cards | Timing of ART initiation |
| b. retention in care 90 days postpartum | ART register | Numerator:HIV-Infected |

| | | |
|---|---|---|
| | CTC2 cards<br>CHW register | women retained in care at 90 days postpartum<br>Denominator: HIV-infected pregnant women |
| c. adherence to ART 90 days postpartum | Pharmacy logbook | ART adherence (MPR) |
| 2. Impact heterogeneity by intensity of exposure to CHWs | CHW logs | Number of CHW visits to individual women |
| 3. Acceptability and feasibility | ANC register | ≥4 ANC visits<br>facility delivery<br>postnatal visit attendance<br>EID testing<br>Denominator: HIV-infected postpartum women |

*7.3 Sampling and Data Collection*

For many of the impact and process outcomes, we will use ANC logs to abstract data from the women who will comprise the denominator (e.g., proportion of women retained in care 90 days postpartum). Once we identify these women through the health records, we can use their medical ID to find CHW logs. The logs will provide us with further details of the services provided to each woman.

In some health facilities, electronic medical records will be available and we will not have to sample a subset of women. However, in others the records will be fully paper based and we will have to sample women for data abstraction. In health facilities where there are <50 HIV-infected pregnant or postpartum women in the baseline or endline cohorts (determined with the CHW log and ANC register), we will abstract data from the ART register on *all* women. At health facilities where there are ≥50 HIV-infected pregnant or postpartum women in the cohorts, we will abstract a predetermined sampling fraction such that 75% of women are sampled from cohorts with 50-100 women, 50% of women are sampled from cohorts with 100-200 women, etc. This approach will ensure that the sample size is adequate to detect a minimum increase in retention in care at 90 days postpartum. In facilities where data are manually abstracted from paper records, we will use Qualtrics Offline Surveys on Android tablets.

In facilities with 50 or more HIV-infected pregnant women in the baseline or endline cohorts, we will use a systematic random sampling procedure with a random start. Research assistants will select a number from a random number table and then select the woman in this position for the study. The research assistant will then sample every *k* eligible woman thereafter, where *k* is the sampling fraction, described above.

*7.4. Evaluation Question #1:* What is the impact of integrating community-based MNCH and PMTCT services using CHWs in Shinyanga, Tanzania on:

a. The timing of ART initiation among HIV-infected pregnant and postpartum women;
b. Retention of HIV-infected women in care 90 days postpartum; and
c. Adherence to ART at 90 days postpartum.

All three indicators will be measured primarily from ANC records, though we will also back check these records with CHW logs to ensure accuracy and quality of the data. We will analyze all three measures through difference in difference strategyin order to capture differences in outcome between the control and treatment groups.

*7.5. Evaluation Question #2: Is the impact of integrating community-based MNCH and PMTCT*

*services on retention in care and adherence greater among women who had more contact with CHWs?*

To answer this question, we will monitor the CHW logs to measure (a) the duration of visits with CHWs and (b) the frequency of visits (per week, per month). We will then compare the frequency and duration of visits to each woman's retention in the program, and their level of participation (measured through adherence to ART) to find the correlation between CHW contact and participation.

*7.6. Evaluation Question #3: Is integration of community-based MNCH and PMTCT services acceptable and feasible for CHW, health facility staff, and pregnant and postpartum women, and are there potential spillover benefits on other maternal health outcomes?*

We will conduct a mixed methods process evaluation, including quantitative analysis of process outcomes feedback from stakeholders (two facility personnel, five CHWs, five clients, two Amref staff)), and in-depthinterviews with women, in order to better understand the effectiveness and feasibility of the intervention. We will focus our efforts on answering the following key questions:

a. Was the intervention implemented as intended? Was there heterogeneity in implementation? Were standard operating protocols followed consistently and correctly?
b. Were there community- or facility-level factors that influenced the success of the intervention? Was the intervention impeded by the time availability of CHW or facility staff?
c. Did the intervention motivate pregnant and postpartum women? How? What aspects were motivating and why? Did the intervention inadvertently increase stigma?
d. What were successful or challenging aspects of the intervention throughout the causal chain (***Figure 1***)? What could be improved?

The interview and focus guides will be developed to align with constructs from the theory of change to understand how the intervention works or does not work. For example, we will ask women about their perceptions of the process outcomes (ANC, facility delivery, postpartum visit attendance) before and after their pregnancy and how the Action Birth Card did or did not influence their behavior to engage/not engage in these services. Furthermore, among HIV infected women, we will probe about the process of initiating ART, coming to terms with lifelong treatment, PMTCT, and how they think about remaining on ART after delivery. We will then apply the lens of the intervention to understand whether the ABC or CHW interaction altered their expectations, preferences, or behaviors. Together, these data will help to triangulate our results from the quantitative analysis.

## 8. ETHICAL CONSIDERATIONS

This evaluation has been approved by ethical review boards at the University of California, Berkeley, conditional on approval by the Amref Health Africa Tanzania review board.

A primary concern the study team has faced has been to ensure the data collection maintains the anonymity of patients. In order to ensure this in the quantitative of the study, we will abstract medical record information from randomly sampled women. Information will come from medical and pharmacy records, and CHW logs. We will identify patients by their ANC ID number in order to pool data from these various sources. Once merged, we will drop identifying information (clinic ID, birthdate). Other demographic information will be retained (age in years,

ethnicity, education, etc.), but none of these variables will be identifiable without the clinic ID or birthdate.

In the qualitative evaluation, we will only collect basic demographic information in order to ensure diversity in the study population (e.g., sex, age in years, year of HIV diagnosis and year of ART initiation, if applicable). Interviews will be audio recorded, and the audio files and transcripts will be identified with a number only for record keeping purposes. The number will not be used to link particular findings back to an individual participant. Audio files, once transcribed, will be securely stored and later destroyed (one year after the study's conclusion).

## 9.LIMITATIONS

As with any study design, ours is not without limitations. Because the control and treatment groups will be measured at different times, time may be a confounding factor to the analysis and a threat to out internal validity. We minimize this effect, however, by measuring information about the women at a similar point in time in their pregnancy (90 days postpartum). We do not anticipate significant issues in validity due to the time period of data collection.

## 10. ABBREVIATIONS

ANC = Antenatal care
ART = Antiretroviral therapy
ARV = Antriretrovirals
CHW = community health worker
eMTCT = elimination of mother-to-child transmission
MNCH = maternal, newborn and child health services
PMTCT = Prevention of mother to child transmission
RCH = Reproductive and child health

## References


1. World Health Organization. Accountability for Women's and Children's Health: Recommendation 2: Health indicators. http://www.who.int/woman_child_accountability/progress_information/recommendation2/en/. Accessed July 3, 2014.
2. UNAIDS. Report on the Global AIDS Epidemic. Geneva2013.
3. Joint United Nations Programme on HIV/AIDS (UNAIDS). *Global Plan Towards the Elimination of New HIV Infections Among Children By 2015 and Keeping their Mothers Alive, 2011-2015.* Geneva2011.
4. World Health Organization. *Antiretroviral Drugs for Treating Pregnant Women and Preventing HIV Infection in Infants: Recommendations for a public health approach.* Geneva2010.
5. Sibanda EL, Weller IV, Hakim JG, Cowan FM. The magnitude of loss to follow-up of HIV-exposed infants along the prevention of mother-to-child HIV transmission continuum of care: a systematic review and meta-analysis. *Aids.* Nov 13 2013;27(17):2787-2797.
6. Paintsil E, Andiman WA. Update on successes and challenges regarding mother-to-child transmission of HIV. *Curr Opin Pediatr.* 2009;21:94-101.
7. National Bureau of Statistics (NBS). *Tanzania HIV/AIDS and Malaria Indicator Survey, 2011-12.* Dar es Salaam: Tanzania Commission for AIDS (TACAIDS);2013.
8. Ministry of Health and Social Welfare. Tanzania PMTCT Partners Catalogue. 2013; http://pmtct.or.tz/wp-content/uploads/2013/01/TZ_A5_Catalogue_-Revised_November_2013.pdf. Accessed 21 August 2014.
9. World Health Organization. *Programmatic Update. Use of Antiretroviral Drugs for Treating Pregnant Women And Preventing HIV Infection in Infants.* Geneva2012.
10. Centers for Disease C, Prevention. Impact of an innovative approach to prevent mother-to-child transmission of HIV--Malawi, July 2011-September 2012. *MMWR. Morbidity and mortality weekly report.* Mar 1 2013;62(8):148-151.
11. Tenthani L, Haas AD, Tweya H, et al. Retention in care under universal antiretroviral therapy for HIV-infected pregnant and breastfeeding women ('Option B+') in Malawi. *Aids.* Feb 20 2014;28(4):589-598.
12. Nachega JB, Uthman OA, Anderson J, et al. Adherence to antiretroviral therapy during and after pregnancy in low-income, middle-income, and high-income countries: a systematic review and meta-analysis. *Aids.* Oct 23 2012;26(16):2039-2052.
13. World Health Organization, UNICEF, Interagency Task Team of Prevention of HIV Infection in Pregnant Women M, and their Children,. *Guidance on Global Scale-Up of the Prevention of Mother to Child Transmission of HIV.* Geneva2007.
14. UNAIDS. Report on the Global AIDS Epidemic. Geneva2012.
15. Suthar AB, Rutherford GW, Horvath T, Doherty MC, Negussie EK. Improving antiretroviral therapy scale-up and effectiveness through service integration and decentralization. *Aids.* Mar 2014;28 Suppl 2:S175-185.
16. Joint United Nations Program on HIV/AIDS (UNAIDS), President's Emergency Plan for AIDS Relief (PEPFAR). *Task Shifting: Global Recommendations and Guidelines.* Geneva: World Health Organization;2008.
17. The President's Emergency Plan for AIDS Relief (PEPFAR). PEPFAR Guidance on Integrating Prevention of Mother to Child Transmission of HIV, Maternal, Neonatal, and Child Health and Pediatric HIV Services. 2011; http://www.pepfar.gov/documents/organization/158963.pdf. Accessed September 11, 2014.

18. Killam WP, Tambatamba BC, Chintu N, et al. Antiretroviral therapy in antenatal care to increase treatment initiation in HIV-infected pregnant women: a stepped-wedge evaluation. *Aids.* Jan 2 2010;24(1):85-91.
19. Mwai GW, Mburu G, Torpey K, Frost P, Ford N, Seeley J. Role and outcomes of community health workers in HIV care in sub-Saharan Africa: a systematic review. *Journal of the International AIDS Society.* 2013;16(1):18586.
20. Chang LW, Kagaayi J, Nakigozi G, et al. Effect of peer health workers on AIDS care in Rakai, Uganda: a cluster-randomized trial. *PLoS One.* 2010;5(6):e10923.
21. Torpey KE, Kabaso ME, Mutale LN, et al. Adherence support workers: a way to address human resource constraints in antiretroviral treatment programs in the public health setting in Zambia. *PLoS One.* 2008;3(5):e2204.
22. Igumbor JO, Scheepers E, Ebrahim R, Jason A, Grimwood A. An evaluation of the impact of a community-based adherence support programme on ART outcomes in selected government HIV treatment sites in South Africa. *AIDS Care.* Feb 2011;23(2):231-236.
23. Zachariah R, Teck R, Buhendwa L, et al. Community support is associated with better antiretroviral treatment outcomes in a resource-limited rural district in Malawi. *Trans R Soc Trop Med Hyg.* Jan 2007;101(1):79-84.
24. Jaffar S, Amuron B, Foster S, et al. Rates of virological failure in patients treated in a home-based versus a facility-based HIV-care model in Jinja, southeast Uganda: a cluster-randomised equivalence trial. *Lancet.* Dec 19 2009;374(9707):2080-2089.
25. Hsieh A, Rodrigues J, Skovdal M, et al. From patient to person: the need for an 'HIV trajectories' perspective in the delivery of prevention of mother-to-child-transmission services. *Aids.* Jul 2014;28 Suppl 3:S399-409.
26. le Roux IM, Tomlinson M, Harwood JM, et al. Outcomes of home visits for pregnant mothers and their infants: a cluster randomized controlled trial. *Aids.* Jun 1 2013;27(9):1461-1471.
27. Tomlinson M, Doherty T, Ijumba P, et al. Goodstart: a cluster randomised effectiveness trial of an integrated, community-based package for maternal and newborn care, with prevention of mother-to-child transmission of HIV in a South African township. *Trop Med Int Health.* Mar 2014;19(3):256-266.
28. Kim MH, Ahmed S, Buck WC, et al. The Tingathe programme: a pilot intervention using community health workers to create a continuum of care in the prevention of mother to child transmission of HIV (PMTCT) cascade of services in Malawi. *Journal of the International AIDS Society.* 2012;15 Suppl 2:17389.
29. Lewin S, Munabi-Babigumira S, Glenton C, et al. Lay health workers in primary and community health care for maternal and child health and the management of infectious diseases. *Cochrane database of systematic reviews.* 2010(3):CD004015.
30. Pangaea Global AIDS Foundation, Clinton Health Access Initiative. *Tanzania: The Reproductive Child Health (RCH) Platform Approach.* Oakland, California2013.
31. UNICEF Health Section, Program Division. *Access to healthcare through comunity health workers in East and Southern Africa.* New York: UNICEF;2014.
32. McMahon JH, Jordan MR, Kelley K, et al. Pharmacy adherence measures to assess adherence to antiretroviral therapy: review of the literature and implications for treatment monitoring. *Clin Infect Dis.* Feb 15 2011;52(4):493-506.
33. Messou E, Chaix ML, Gabillard D, et al. Association between medication possession ratio, virologic failure and drug resistance in HIV-1-infected adults on antiretroviral therapy in Cote d'Ivoire. *J Acquir Immune Defic Syndr.* Apr 2011;56(4):356-364.
34. Goldman JD, Cantrell RA, Mulenga LB, et al. Simple adherence assessments to predict virologic failure among HIV-infected adults with discordant immunologic and clinical

responses to antiretroviral therapy. *AIDS Res Hum Retroviruses.* Aug 2008;24(8):1031-1035.
35. Hong S, Nachega J, Jerger L, et al. Medication Possession Ratio Predictive of Short-term Virologic and Immunologic Response in Individuals Initiating ART: Namibia. 19th Conference on Retroviruses and Opportunistic Infections; 2012; Seattle.
36. World Health Organization. *Antenatal Care Randomized Trial: Manual for the Implementation of the New Model.* Geneva2002.
37. Andersen C, Njau PF, McCoy SI. Loss to Follow-up Among Option B+ Patients in Shinyanga Region. Unpublished.2014.
38. National Bureau of Statistics (NBS) [Tanzania] and ICF Macro. *Tanzania Demographic and Health Survey 2010.* Dar es Salaam, Tanzania: NBS and ICF Macro;2011.
